# Scale-free behavioral cascades and effective leadership in schooling fish

**DOI:** 10.1038/s41598-022-14337-0

**Published:** 2022-06-24

**Authors:** Julia Múgica, Jordi Torrents, Javier Cristín, Andreu Puy, M. Carmen Miguel, Romualdo Pastor-Satorras

**Affiliations:** 1 Departament de Física, Universitat Politècnica de Catalunya, Campus Nord B4, 08034 Barcelona, Spain; 2 Departament de Física de la Matèria Condensada, Universitat de Barcelona, Martí i Franquès 1, 08028 Barcelona, Spain; 3 Istituto Sistemi Complessi, Consiglio Nazionale delle Ricerche, UOS Sapienza, 00185 Rome, Italy; 4 Dipartimento di Fisica, Universita’ Sapienza, 00185 Rome, Italy; 5 Universitat de Barcelona Institute of Complex Systems (UBICS), Universitat de Barcelona, Barcelona, Spain

**Keywords:** Biological physics, Nonlinear phenomena, Phase transitions and critical phenomena, Animal behaviour

## Abstract

Behavioral contagion and the presence of behavioral cascades are natural features in groups of animals showing collective motion, such as schooling fish or grazing herbivores. Here we study empirical behavioral cascades observed in fish schools defined as avalanches of consecutive large changes in the heading direction of the trajectory of fish. In terms of a minimum turning angle introduced to define a large change, avalanches are characterized by distributions of size and duration showing scale-free signatures, reminiscent of self-organized critical behavior. We observe that avalanches are generally triggered by a small number of fish, which act as effective leaders that induce large rearrangements of the group’s trajectory. This observation motivates the proposal of a simple model, based in the classical Vicsek model of collective motion, in which a given individual acts as a leader subject to random heading reorientations. The model reproduces qualitatively the empirical avalanche behavior observed in real schools, and hints towards a connection between effective leadership, long range interactions and avalanche behavior in collective movement.

## Introduction

Collective motion is an ubiquitous phenomenon in nature, observed in a wide variety of different living systems and on an even wider range of scales, from mammal herds and fish schools, to bacteria colonies and cellular migrations^1–3^. The study of collective motion allows scientists to infer the intricate interaction mechanisms governing the diversity of behaviors found in natural grouping species^1,4–6^. Identifying the most relevant traits will prove essential if we ever want to take advantage of nature wisdom for engineering applications such as in swarm robotics^7^ or in driver-less cars. Social animals group and travel together to gain several benefits, from better foraging and more efficient offspring training, to improved navigational accuracy and reduced risk of predation^8^. Examples illustrating the emergence of ordered collective motion in social animal groups can take the spectacular form of wildebeest herds crossing deserts in Africa, or huge fish schools running away coordinately from predators^9^. From a more mundane perspective, the seemingly simple movement of a sheep herd crossing a road also arises as a result of the collective, coordinated motion of individual sheep^10^.

A common view of collective motion, implemented in most numerical models, is that coherent spatio-temporal patterns emerge spontaneously from decentralized interactions among identical self-propelled group members^1^. The kind of coordination required to produce such impressive patterns, however, requires an efficient transfer of information among the group components. In this regard, leadership is sometimes brought about to rationalize the cooperative movements of animal groups by single individuals that appear to have a strong influence on the flock behavior^11^. The effects of leadership have been considered in several contexts, including crowd behavior^12^, hierarchical leadership^13^, linear response theory in flocking systems^14,15^ or the emergence of complex patterns of cooperation and conflict^16^. Leadership can arise as a natural instinct in some animals, which form a permanent hierarchical structure, but in other cases it can exhibit a switching dynamics that can even depend on context^5,17–19^. In this sense, effective leadership can come from individuals having useful information about their environment, such as the position of food or predators, not visible to the rest of the flock^20,21^. Another important aspect of collective animal motion is the existence of spontaneous individual-level behavioral variations, which may be transmitted to the group as if those particular individuals were effective group leaders. As a result of abrupt changes of dynamic behavior at the level of one or a few individuals, animal groups can exhibit intermittent collective rearrangements, or can even undergo state transitions at the macroscopic level. Collective behavioral oscillations or waves in groups have been reported in golden shiners^4^, which were related to an underlying or *hidden* communication network^4^. On the other hand, sheep herds have been shown to pass from slow group dispersive motion while grazing, to rapid aggregation induced by sudden, individual changes of speed^10^ in the absence of nearby threatening sources. Most interestingly, these experimental studies emphasize that animal rearrangements can either spread extensively within the group or extinguish rapidly, leading to an avalanche-like type of response with a broad-tailed distribution of avalanche magnitudes^4,10^. This sort of avalanche behavior is well known in the physics literature^22^, where it has been discussed in magnetic materials^23^, superconductors^24^, plastic deformation of crystalline materials^25^, fracture phenomena^26^, or earthquakes^27^.

In this paper we examine the interplay between effective leadership and behavioral cascades (avalanching behavior) by means of an empirical analysis of the movement of black neon tetra fish *Hyphessobrycon herbertaxelrodi*, and through the theoretical analysis of a variation of the classical Vicsek model^28^ that includes an explicit leader. In our empirical analysis, we define avalanches in terms of changes in the fish heading above a given turning angle threshold, which lead to a sudden reorientation of the global trajectory of the school. We observe that the distributions of size and duration of the measured avalanches show scale-free signatures in analogy with self-organized critical processes^29^ that can be described in terms of a set of characteristics scaling exponents. We explore the possible presence of leadership by considering the statistics of avalanche initiators, observing that some fish have an anomalous large probability of starting an avalanche, acting thus as effective leaders promoting substantial school rearrangements. In order to check the general effects of leadership in avalanche behavior, we consider a Vicsek-like model in which a global leader, which exerts a long range influence over the group members, alternates a directed motion, unaffected by other individuals, with sudden variations of its direction of motion, in the spirit of run-and-tumble locomotion^30^. Akin to our experimental observations, the model exhibits intermittent scale-free avalanche-like behavior, not present in the original model. Our results confirm the presence of scale-free signatures in behavioral cascades in collective motion^4^ and highlight the role of effective leadership and long range interactions in the emergence of this sort of collective behavior.

## Results

### Empirical analysis of schooling fish

We have analyzed the avalanche behavior in black neon tetra (*Hyphessobrycon herbertaxelrodi*), a small freshwater fish (adult mean body size of 2.5 cm) that have a strong tendency to form compact and highly polarized schools^31^. Experiments, performed by the group of F. S. Beltrán and V. Quera at the Institute of Neurosciences, University of Barcelona (Spain), consisted in groups of 40 individuals, freely swimming in an experimental rectangular tank of dimensions $100 \times 93$ cm and 5 cm of depth. Videos of the fish movement were recorded at 20 frames per second with a resolution of $1072 \times 1004$ pixels. Three independent recordings, each of length $T=12{,}000$ frames were performed. The path of individual fish was digitized using a custom-made tracking software. The paths obtained were later visually inspected to correct for a few anomalously large jumps, due to the switching of fish identities in the tracking process. The trajectories obtained were finally smoothed by applying a Savitzky-Golay filter of window length 7 and polynomial order 3^32^. The final result were three digital data series, labeled A, B and C, that we analyzed numerically.

#### Avalanche analysis

Supplementary Video SV 1 shows a rendering of a segment of the school evolution in time for series A. The heading of each fish, marked by a short arrow, is defined in terms of its instantaneous velocity $\mathbf {v}_i(t)$. Given the path of a fish as a function to time $\mathbf {r}_i(t)$, $t=1, 2, \ldots , T$ (time measured in frames), we define the velocity at time *t* using a Richardson extrapolation of order 4, in terms of the expression^33^

1 $$\begin{aligned} \mathbf {v}_i(t) = \frac{1}{12} \left[ \mathbf {r}_i(t-2) - 8 \mathbf {r}_i(t-1) + 8 \mathbf {r}_i(t+1) - \mathbf {r}_i(t+2) \right] , \qquad t = 3, 4, \ldots , T-2. \end{aligned}$$

As we can see in Supplementary Video SV 1, fish tend to move in a coherent and highly polarized fashion, swimming with a common and slowly changing average velocity. In this regime, heading variations are small and rather smooth. However, at some instants of time, we can recognize swift rearrangements of the individuals’ headings, that lead to a change of the average orientation of the school, accompanied by an increase of the average velocity and a decrease and a delayed increase of the global order of the school (see the animated plot in SV 1). We interpret these sudden rearrangements of individual heading as triggerers of *avalanches* of activity. Avalanches are triggered at different positions within the experimental tank and, in particular, they are also initiated near the tank’s walls, but they are not restricted to occur always there. In order to quantify them, we fist examine the angular variations in the heading of individual fish, defined as the *turning angle* $\varphi _i(t)$ formed by the velocity vectors $\mathbf {v}_i(t+1)$ and $\mathbf {v}_i(t)$, and computed as

2 $$\begin{aligned} \varphi _i(t) = \left|\arctan \left\{ \frac{\Vert \mathbf {v}_i(t) \times \mathbf {v}_i(t+1) \Vert }{\Vert \mathbf {v}_i(t) \cdot \mathbf {v}_i(t+1) \Vert } \right\} \right|, \end{aligned}$$

where $\times$ stand for the vectorial product and $\Vert \cdot \Vert$ represents the vector modulus. For symmetry reasons, the angles, computed in the interval $[-\pi , \pi ]$, are projected onto $[0, \pi ]$.

**Figure 1:**
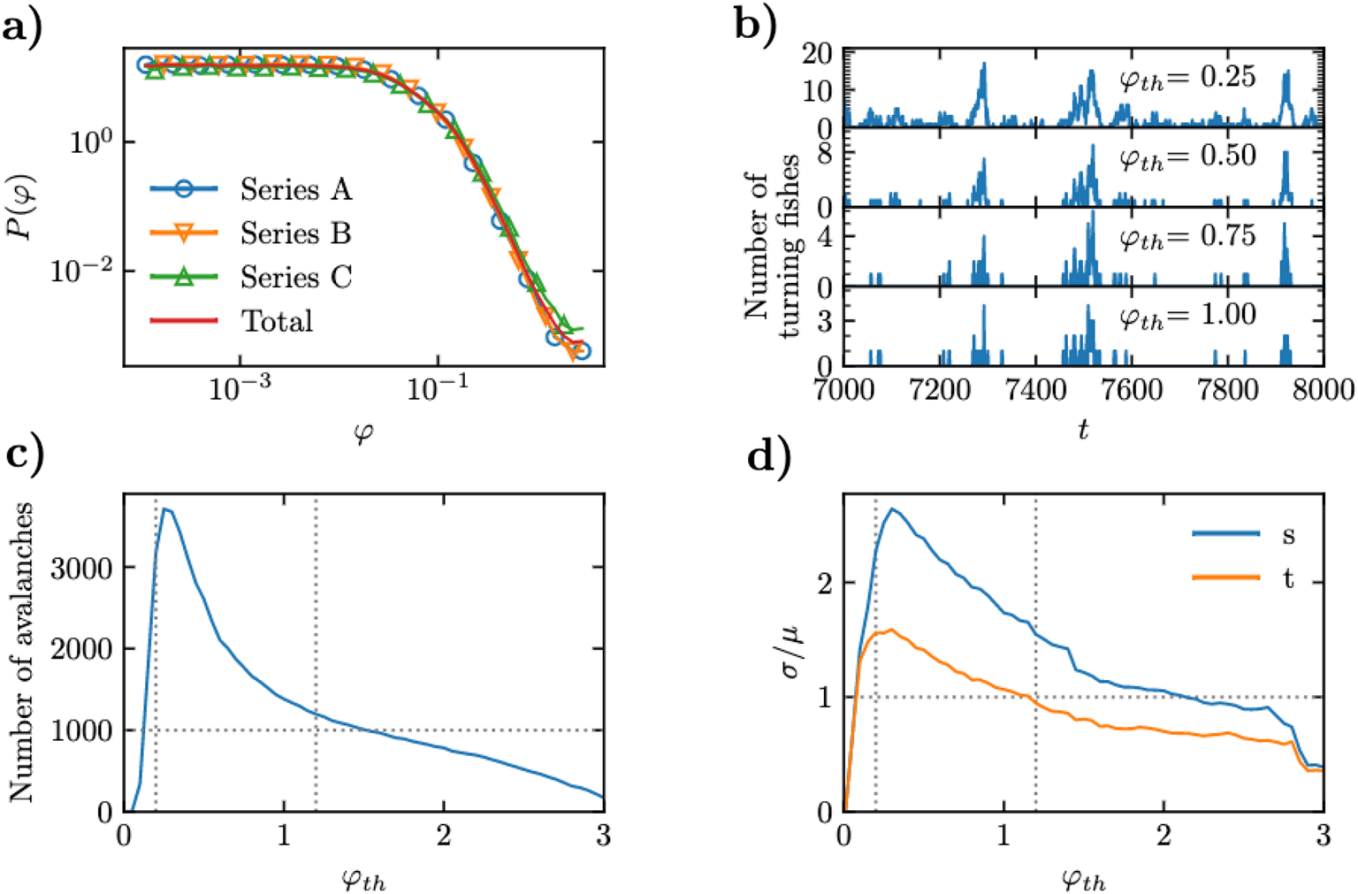
(**a**) Probability density of turning angles $\varphi$ of individual fish. (**b**) Number of fish turning an angle larger than $\varphi _\mathrm {th}$ in a sequence of 1000 frames in data series A, for different values of the turning threshold. (**c**) Number of avalanches observed as a function of the turning threshold. (**d**) Relative fluctuations of the avalanche size (*s*) and duration (*t*) distributions as a function of the turning threshold. Vertical lines in (**c**) and (**d**) represent the turning threshold interval [0.2, 1.2].

In Fig. 1a we plot the distribution of turning angles $P(\varphi )$ for the different data series (hollow symbols) and for the aggregation of all three of them (red line). Discarding some extremely small values of $\varphi$, which can be attributed to imprecision of the tracking algorithm when following an essentially straight segment, the distributions show an extended plateau for small turning angles, corresponding to stretches of time in which fish barely change their heading and are thus compatible with movement along a smoothly winding trajectory. Instead, for values larger than 0.01 radians, the distribution starts to decrease sharply. These large turns correspond to the sparsely distributed large rearrangements of direction observed in SV 1. In order to quantitatively identify avalanches, as is customarily done in condensed matter physics^34^, we define a *turning threshold* $\varphi _\mathrm {th}$ that distinguishes small turns $\varphi < \varphi _\mathrm {th}$, associated to smooth trajectories, from large turns $\varphi > \varphi _\mathrm {th}$, associated to sudden rearrangements that trigger an avalanche. In Fig. 1b we plot, for a given value of the threshold, the number of *active* fish, defined as those performing a turn larger than $\varphi _\mathrm {th}$, as a function of time. Here we can see the actual presence of turning avalanches, defined as trains of consecutive frames in which more than one fish is active, delimited by two frames (one at the beginning and another at the end of the train) with no active fish. These curves highlight the intermittent and heterogeneous character of avalanches, which may be rather small or can also reach relatively large sizes.

Our experimental data only allows the identification of a limited number of avalanches. Indeed, in Fig. 1c we plot the total number of recorded avalanches as a function of the turning threshold. From here we observe that the range of values of $\varphi _\mathrm {th}$ that lead to at least 1000 avalanches range approximately in the interval [0.20, 1.50]. To study the statistics of avalanches, we compute their duration *t* and size *s*, defined as the number of consecutive time steps (frames) with at least one active fish, and the sum of the number of active fish at each time step of an avalanche, respectively. Notice that, since a fish can be active in more than one step along the duration of an avalanche, the avalanche size *s* is in general larger than the avalanche duration *t*, and can be larger than the total number of fish in the experiment. A first broad statistical characterization of avalanches is given by the relative size and duration fluctuations, measured as the standard deviation $\sigma$ divided by the corresponding average value $\mu$. In Fig. 1d, we plot these relative fluctuations for both *s* and *t*, respectively. From this plot, we observe that relative fluctuations are only larger than 1 for threshold values within the interval between 0.1 and 1.2. We therefore restrict our analysis to the conservative threshold interval [0.20, 1.20].

We consider the shape of the probability distributions of avalanche sizes, *P*(*s*), and durations, *P*(*t*), focusing on the cumulative distributions,

3 $$\begin{aligned} P_c(s) = \sum _{s' = s}^\infty P(s') \qquad \text{ and } \qquad P_c(t) = \sum _{t' = t}^\infty P(t'). \end{aligned}$$

**Figure 2:**
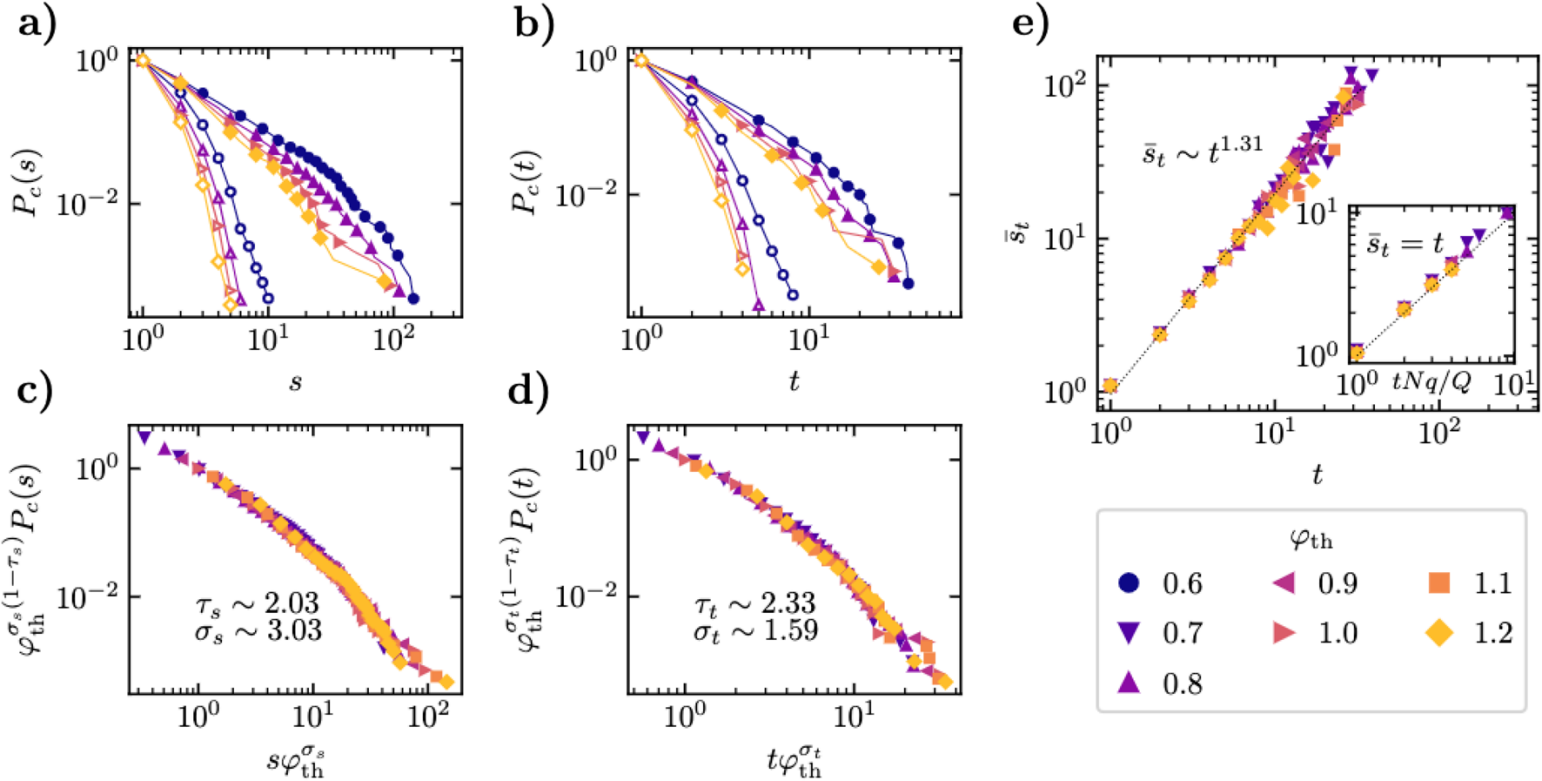
(**a**) Cumulative probability distribution of the avalanche sizes $P_c(s)$ for different values of the turning threshold $\varphi _\mathrm {th}$. (**b**) Cumulative probability distribution of the avalanche durations $P_c(t)$ for different values of the turning threshold $\varphi _\mathrm {th}$. In (**a**) and (**b**), filled symbols represent the actual empirical distributions, while hollow symbols correspond to distributions obtained by randomizing the turning angles in the trajectory of each fish. (**c**) Check of the scaling of the cumulative size distribution with the turning threshold, as given by Eq. (5). (**d**) Check of the scaling of the cumulated time distribution with the turning threshold, as given by Eq. (5). (**e**) Average size $\bar{s}_t$ of avalanches of fixed duration *t* as a function of *t*. The main plot shows the empirical data. The inset presents the results from a randomization of the turning angles in each fish trajectory. In this case, we plot the average duration as a function of the theoretical prediction *Nqt*/*Q*, see Eq. (13).

In Fig. 2a we plot in full symbols the cumulative size distribution obtained for different values of $\varphi _\mathrm {th}$. From the double logarithmic scale in the plot, we can see that the size distributions show long tails, compatible with a power-law behavior of the form $P(s) \sim s^{-\tau _s}$ for small values of *s*. This power-law behavior is due to the correlated nature of turns in the fish school, a feature that can be corroborated from the comparison of these results with the avalanche distributions obtained from trajectories reconstructed by randomizing the sequence of turning angles of each fish. In the latter case, one obtains a clear exponential decay, as shown in hollow symbols in Fig. 2a,b; see “Methods” for an analytical derivation.

Upon closer scrutiny, we can also observe that, for sufficiently large $\varphi _\mathrm {th}$, the initial power-law behavior of the size distributions is followed by a faster decay for *s* larger than a characteristic size $s_c$ that appears to be a decreasing function of the threshold $\varphi _\mathrm {th}$. Inspired by the observations in other avalanche systems^35^ and in models of self-organized criticality^29^, we can assume that, for different values of the threshold, the size distributions exhibit a scaling behavior of the form

4 $$\begin{aligned} P(s) = s^{-\tau _s} G_s\left( \frac{s}{s_c(\varphi _\mathrm {th})}\right) \end{aligned}$$

where the scaling function $G_s(z)$ is constant for small $z\ll 1$ and decays rapidly to zero for $z \gg 1$. In analogy with avalanches in condensed matter and critical phenomena^36,37^ we make the ansatz for the behavior of the size cut-off $s_c(\varphi _\mathrm {th}) \sim \varphi _\mathrm {th}^{-\sigma _s}$, where $\sigma _s$ is a characteristic exponent. We can estimate the values of the exponents by noticing that Eq. (4) implies, for the cumulative distribution, $P_c(s) = s^{-\tau _s + 1} F_s \left( s \varphi _\mathrm {th}^{\sigma _s} \right)$, where $F_s(z)$ is another scaling function. The previous expression can be rewritten as

5 $$\begin{aligned} \varphi _\mathrm {th}^{\sigma _s(1-\tau _s) }P_c(s) = F'_s \left( s \varphi _\mathrm {th}^{\sigma _s} \right) , \end{aligned}$$

where $F'_s(z) = z^{-\tau _s +1} F_s(z)$. Equation (5) implies that, when plotting the rescaled distribution $\varphi _\mathrm {th}^{\sigma _s(1-\tau _s) } P_c(s)$ as a function of the rescaled size $s \varphi _\mathrm {th}^{\sigma _s}$, with the correct exponents $\tau _s$ and $\sigma _s$, plots for different values of $\varphi _\mathrm {th}$ should collapse onto the same universal function $F'_s(z)$. Using this idea, one can estimate numerically the exponents $\tau _s$ and $\sigma _s$ as those that provide the best collapse of the data rescaled using Eq. (5) for the different values of $\varphi _\mathrm {th}$, see “Methods”.

Following this approach, using values of $\varphi _\mathrm {th}\in \{ 0.7, 0.8, 0.9, 1.0, 1.1, 1.2\}$, we estimate the exponents $\tau _s \simeq 2.03$ and $\sigma _s \simeq 3.03$. In Fig. 2c we show the data collapse for Eq. (5) obtained for the cumulated size distributions using these values. Different intervals of the turning threshold provide slightly different values of the exponents, from which we estimate the average exponents quoted in Table 1. The same procedure can be applied to the duration distribution, see Fig. 2b, where now the cumulative duration distribution $P_c(t)$ fulfills Eq. (5) with the corresponding exponents $\tau _t$ and $\sigma _t$. In the same interval of thresholds we find $\tau _t \simeq 2.33$ and $\sigma _t \simeq 1.59$, see Fig. 2d, while the average exponents are given in Table 1. We can check the validity of these results considering that, for small values of *s* and *t*, the distributions $P(s) \sim s^{-\tau _s}$ and $P(t) \sim t^{-\tau _t}$ imply that the average size of avalanches of duration *t*, $\bar{s}_t$, takes the form

6 $$\begin{aligned} \bar{s}_t \sim t^{m}, \;\;\; \mathrm {with} \;\;\; m = \frac{\tau _t -1}{\tau _s -1}. \end{aligned}$$

In Fig. 2e we represent the empirical average avalanche size $\bar{s}_t$ as a function of the duration *t*. For the different values of the turning threshold considered, we estimate numerically that $\overline{s} \sim t^{1.31}$. This observation is in good agreement with the expression in Eq. (6), which, using the values from Table 1, yields $m = 1.4(2)$. In Fig. 2e we also show the average avalanche size observed in randomized avalanches, which shows a linear dependence as expected theoretically, see “Methods”. This last result highlights the relevant effect of turning angle correlations in real fish.

**Table 1:** Summary of scaling exponents for the avalanche size and duration distributions obtained from observations of a real fish school and from the Vicsek model with a perturbed leader.

	Schooling fish			
	$\tau _s$	$\sigma _s$	$\tau _t$	$\sigma _t$
	2.0(1)	3.1(3)	2.4(1)	1.70(4)

	Vicsek model with a perturbed leader $\varphi _\mathrm {th}(\eta ) = 2.5 \pi \eta$			
	$\tau _s$	*D*	$\tau _t$	*z*
$\eta =0.2$	1.73(5)	2.01(2)	4.03(5)	0.39(5)
$\eta =0.3$	1.69(5)	2.01(2)	3.49(5)	0.42(5)

	Vicsek model with a perturbed leader $\varphi _\mathrm {th}(\eta ) = 2.8 \pi \eta$			
	$\tau _s$	*D*	$\tau _t$	*z*
$\eta =0.2$	0.99(5)	2.06(2)	0.26(4)	0.50(5)
$\eta =0.3$	1.04(5)	2.03(2)	0.57(5)	0.52(5)

#### Effective leadership and avalanche behavior

**Figure 3:**
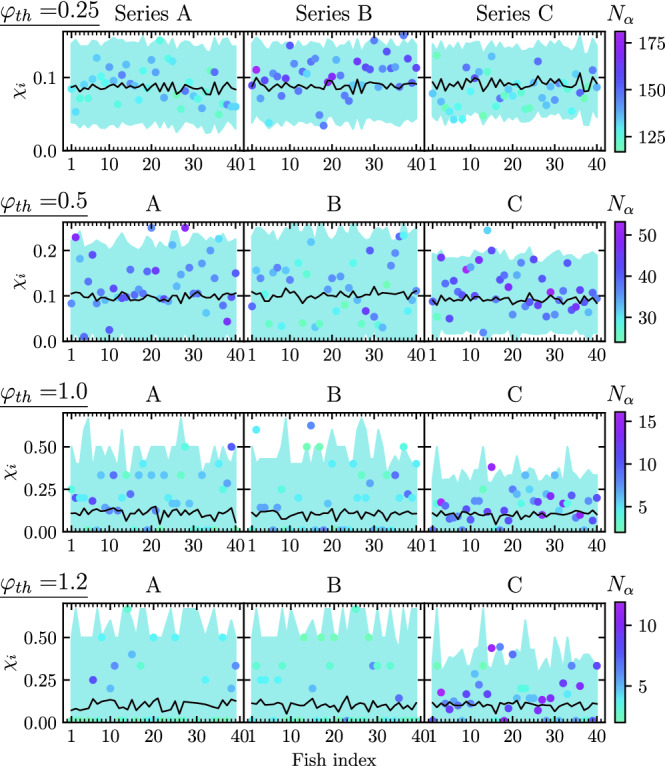
Leadership probability $\chi _i$ computed for each fish *i* in the three data series considered A, B and C columns, left to right) and for different values of the turning threshold $\varphi _\mathrm {th}=0.25, 0.5, 1.0$ and 1.2 (rows, top to bottom). Symbols are color-coded with the number $N_\alpha$ of actual avalanches in which each fish participates. Full lines represent the average leadership probability in a null model of uncorrelated avalanches. The shadowed regions represents the $99\%$ confidence interval of this value.

In order to explain the origin of the avalanche behavior observed in our empirical data, we consider the possibility that avalanches are triggered by some initiator or effective *leader*, which consistently starts the large turning rearrangements that lead to the formation of an avalanche. While several definitions of leadership have been proposed within the field of collective animal motion^38^, here we use a measure explicitly devised to detect the presence of preferential initiators of avalanches. We consider the originator of an avalanche as the fish that performs the first large heading turn in the evolution of the avalanche. As more than one fish can be active in any frame, we consider as initiators all active fish in the first frame of an avalanche. We define the *leadership probability* $\chi _i$ of fish *i* in a given data series as the ratio of the number of avalanches in which the fish *i* is active in the first frame, divided by the total number of avalanches in which fish *i* participates. The calculation is restricted to sufficiently large avalanches, of duration larger than 5 frames. In Fig. 3 we plot the value of $\chi _i$ computed for each one of the $N=40$ fish in each series, for different values of the turning threshold $\varphi _\mathrm {th}$. As we can see, the leadership probability shows an important variation among fish. Moreover, for the largest values of $\varphi _\mathrm {th}$ considered, the leadership probability can take values up to 0.60, indicating that some fish initiate more than half of the avalanches in which they participate.

In order to quantify the relevance of the values of $\chi _i$ obtained, and ascertain that they are not the effect of random fluctuations in the activity of the fish, given our small populations, we compare our empirical estimates with the results obtained in a null model in which the turns performed by fish are completely independent, see “Methods”. The continuous lines in Fig. 3 represent the null model average leadership probability, while the shaded region represents its $99\%$ confidence interval. Our results in Fig. 3 indicate that, with the exception of series C, in all series and for all values of the turning threshold considered, several fish have an unusually very large probability to initiate an avalanche, much larger than the value expected from pure random fluctuations. We can associate them to effective leaders of the school, which initiate with large probability the avalanches in which they participate.

More information can be obtained by considering the evolution of the leadership probability as a function of the turning threshold for each fish in each time series, see Supplementary Fig. (SF) 1. From this plot we can confirm, first of all, that some fish never initiate an avalanche ($\chi _i = 0$) for large values of the turning threshold, while others consistently start much less avalanches than they should by mere random fluctuations. Some other fish behave as initiators for some range of values of the turning threshold. Finally, some fish reliably initiate a large number of avalanches, much more than they should by pure randomness. These fish can be identified as consistent effective leaders, which trigger a large majority of the avalanches, independently of the value of the threshold used to quantitatively define them.

### Modeling avalanches in the presence of leaders

To explore the effects of the presence of leadership in the avalanche behavior of schooling fish, we consider as the simplest modeling scenario a variation of the classic Vicsek model^28^ in which we introduce an effective leader.

#### Model definition

The Vicsek model^28,39^ is defined in terms of *N* self-propelled particles (SPPs), characterized by a position $\mathbf {r}_i$ and a velocity $\mathbf {v}_i$ of constant modulus $v_0$, evolving in a two dimensional space, and thus being fully characterized by the heading angle $\theta _i$ defined by the velocity vector. Particles interact among them by trying to align their instantaneous velocity with the average velocity of the set of nearest neighbors inside a circular region of radius *R* centered in the considered SPP. A noise source of strength $\eta$, representing physical or cognitive difficulties in gathering or processing local information, allows the formation of an ordered (*flocking*) phase at low noise intensities, and of disordered (*swarming*) states at high enough noise values. See “Methods” for further details and simulation conditions.

In the variation we study (see “Methods”) we consider that a given particle, say particle 1, plays the role of an effective global leader with a long range influence over the rest of the SPPs. We notice that long-range interactions have already been considered in models of collective motion, as a mechanism to ensure a compact flock in the absence of periodic boundary conditions^40^. The velocity of the leader, $\mathbf {v}_1(t) = \mathbf {v}_L$ is not affected by the behavior of its neighbors, and therefore its heading remains constant $\theta _1(t) = \theta _L$ over time. The other SPPs can, on the other hand, feel the orienting effect of their local neighborhood as well as that of the leader, independently of their relative distance. Therefore they take it into account when computing the average velocity of their neighbors, to which they try to align.

At this point, it is worth pointing out that this leader can be any individual who first experiences a sudden orientational shift. For this reason we simply assume that its heading will remain constant, and therefore unaffected by its neighbors, until another reorientation of similar characteristics occurs in the system. We have checked that the leader does not need to be always the same individual to obtain the main results of our model. On the other hand, the presence of an unperturbed leader, that is, a leader with a constant heading over time, would have the effect of suppressing the disordered phase exhibited by the classic Vicsek model. As we can see in SF 2, while for the classical model the transition becomes sharper when increasing the systems size *L*, the leader induces an ordered state for any value of $\eta$, with an order parameter (see “Methods”) fairly independent of system size and vanishing only in the limit of maximum noise $\eta =1$.

#### Avalanche behavior in response of leader perturbations

In this section, we focus our attention on the system-wide perturbations that are induced by changes in the preferred direction of motion of the leader. To analyze them, we consider a random reorientation of the leader’s heading by an angle $\Delta \theta _L$, performed in the steady state corresponding to a given value of the noise intensity $\eta$, and measure the subsequent rearrangements that this perturbation induces in the heading of the rest of fish, as given by the turning angle $\varphi _i(t) = \theta _i(t+1) - \theta _i(t)$ projected on the interval $[0, \pi ]$.

**Figure 4:**
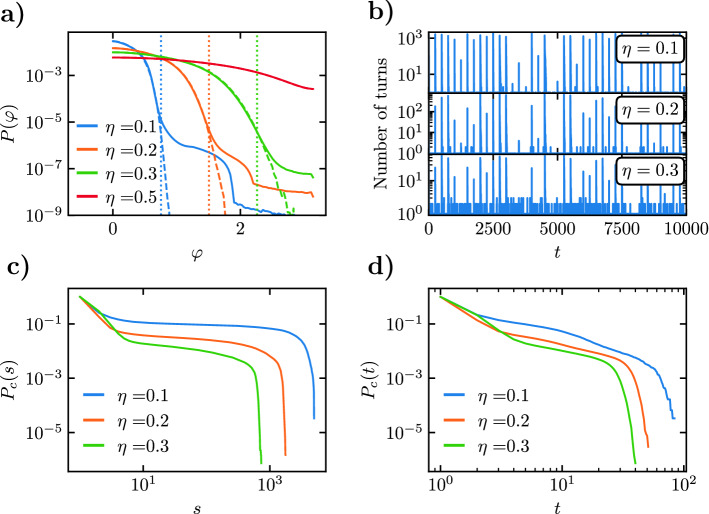
(**a**) Probability density of the turning angles $\varphi$ for the Vicsek model with a leader, for different values of the noise intensity $\eta$, in a system of size $L=220$. Dashed lines correspond to a non-turning leader. Full lines represent a leader perturbed periodically every 250 time steps. Vertical lines, indicating the departure of the distributions for perturbed and non-perturbed leaders, are estimated at a value $\varphi _c(\eta ) \simeq 2.4 \pi \eta$. (**b**) Number of SPPs turning an angle larger than $\varphi _\mathrm {th}(\eta ) = 2.5 \pi \eta$ in a sequence of 10,000 simulation time steps in the Vicsek model with a periodically perturbed leader, for different values of $\eta$. (**c**) Cumulated distribution of sizes $P_c(s)$ of avalanches induced by a periodically perturbed leader in a system of size $L=220$ with turning threshold $\varphi _\mathrm {th}(\eta )$, for different values of the noise intensity. (**d**) Cumulated distribution of durations $P_c(t)$ of avalanches induced by a periodically perturbed leader for different values of the noise intensity.

In Fig. 4a we represent the probability density of SPPs turning angles $P(\varphi )$ in the steady state, for different values of the noise intensity $\eta$. In this plot we consider the model with a fixed, non-turning leader (dashed lines), and the case of a periodically perturbed leader (full lines), in which the leader experiences a random rotation $\Delta \theta _L$ of its heading, uniformly distributed in the interval $[-\pi , \pi ]$, every 250 time steps, a time lapse larger than the maximum avalanche duration recorded in simulations. As we can see, for fixed $\eta$, the two distributions are almost identical for small $\varphi$, while they differ drastically regarding the behavior of the tails beyond a given cut-off turning angle $\varphi _c(\eta )$. A numerical analysis performed for different values of *L* allows to estimate this cut-off as $\varphi _c(\eta ) \simeq 2.4 \pi \eta$. The presence of this turning angle cut-off, not available in empirical data, permits to distinguish the changes of heading due to the effect of the leader perturbations, and suggests that the proper definition of avalanches should consider turning thresholds larger than the cut-off $\varphi _c(\eta )$. In the following, we will fix the value of the threshold to $\varphi _\mathrm {th}(\eta ) = 2.5 \pi \eta$. We notice that for large $\eta \ge 0.5$, the angular distributions with and without perturbations are identical, compatible with a large noise masking external perturbations and making avalanches non discernible.

In Fig. 4b we plot a sample of the number of SPPs that turn an angle larger than $\varphi _\mathrm {th}(\eta )$ as a function of time. This curve emphasizes the heterogeneous character of the avalanche sizes in response to the leader’s changes of direction, akin to what is observed in fish schools: Sometimes a perturbation is followed by a small number of SPPs reorientations; but other times, it triggers the reorientation of a large number of particles. As expected, the strength of the effects of the leader perturbations decreases with increasing noise, indicating that interesting avalanche behavior will only occur for moderate levels of noise.

We compute the cumulative probability distributions $P_c(s)$ and $P_c(t)$, Eq. (3), of observing an avalanche of size and duration larger than *s* and *t*, respectively, plotted in Fig. 4c,d for a turning threshold $\varphi _\mathrm {th}(\eta )$ and different values of $\eta$. As we can see from these plots, the values $\eta =0.2$ and 0.3 lead to size an time distributions analogous to that observed in rearrangement avalanches in real fish schools, with a shape that can be approximated by a power-law form for intermediate values, followed by a crossover to a sharp decrease for large *s* and *t* above a characteristic size or time. The behavior for $\eta =0.1$ is more complex, probably due to the fact that for small noise one expects a fairly homogeneous response with many SPPs following a leader perturbation. We thus discard this value in the following analysis.

**Figure 5:**
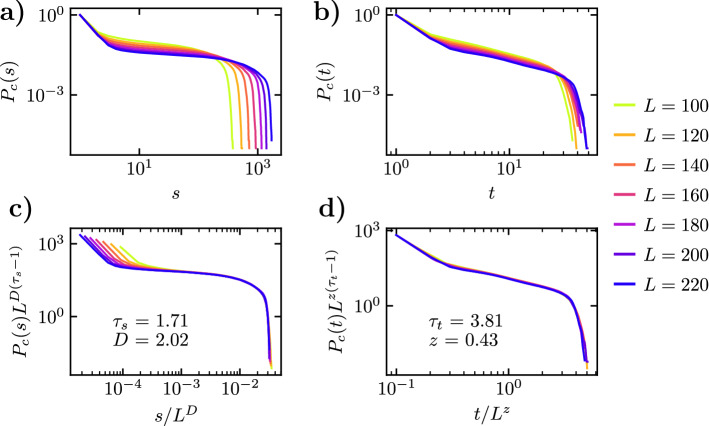
(**a**) Cumulative probability distribution of size $P_c(s)$ of avalanches induced by a perturbed leader in a system with $\eta =0.2$, turning threshold $\varphi _\mathrm {th}(\eta )$ and different values of *L*. (**b**) Cumulative probability distribution of durations $P_c(t)$ of avalanches induced by a perturbed leader. (**c**) Check of the scaling of the cumulated size distribution as given by Eq. (5). (**d**) Check of the scaling of the cumulated time distribution as given by Eq. (5). Statistics are performed over at least $10^5$ different avalanches.

The fact that we work now with a numerical model, allows us to explore the behavior of the system for different systems sizes *L* at a fixed turning threshold, which was not possible in our fixed size empirical data. In Fig. 5a,b we plot the cumulative size and duration distributions in avalanches in the Vicsek model with a turning leader for a turning threshold $\varphi _\mathrm {th}(\eta )$, $\eta =0.2$ and different system sizes. As we can observe, the behavior of the distributions is analogous to that observed in real fish schools, compatible with a power-law decay but that are now truncated by a size and time cut-offs that are functions of the system size *L*. Inspired again by self-organized criticality^29^, we can assume now that the distributions obey a finite-size scaling form

7 $$\begin{aligned} P(s) = s^{-\tau _s} G_s\left( \frac{s}{L^{D}} \right) , \quad P(t) = t^{-\tau _t} G_t\left( \frac{t}{L^z} \right) . \end{aligned}$$

where *D* and *z* are new characteristic exponents that define the characteristic size $s_c(L) \sim L^D$ and time $t_c(L) \sim L^z$ as a function of the system size^29,41^. The better statistics in numerical simulations allow to estimate the characteristic exponents applying the more precise moments analysis technique^42^, see “Methods”. Application of this method leads to the characteristics exponents reported in Table 1. We check the accuracy of these values performing a data collapse analogous to that performed for the avalanches in real fish, which, for the cumulated distributions, takes the form,

8 $$\begin{aligned} L^{D(\tau _s - 1)} P_c(s) = F'_s(s L^D), \qquad L^{z(\tau _t - 1)} P_c(t) = F'_t(t L^z), \end{aligned}$$

see Fig. 5c,d for $\eta =0.2$; the case $\eta =0.3$ is presented in SF 3. As we can see from these values, the exponents show a dependence on the value of the noise $\eta$, although the size exponents appear to be compatible within error bars. It is important to notice that the presence of a rotating leader in necessary to obtain scaling avalanche distributions. Even in the absence of a leader, the heading fluctuations due to noise and interactions in the standard Vicsek model allow to define avalanches for a given threshold. These avalanches, however, show a simple, short ranged exponential distribution, as shown in SF 4.

We have finally checked the effects of changing the turning threshold in the scaling of the distributions as a function of the system size. In SF 5 and SF 6 we show the results for a turning threshold $\varphi _\mathrm {th}= 2.8 \pi \eta$, summarized in Table 1. As we can see, the scaling exponent $\tau _s$ and $\tau _t$ in our model depend on the value of the threshold. This fact is in contrast with the behavior of the fish school, in which the characteristics exponents appear to be independent of the threshold, and thus allowing for a scaling solution of the form given by Eq. (4). Interestingly, the exponents *D* and *z* appear to be rather detail independent, taking the approximate values $D \simeq 2$ and $z \simeq 1/2$ for any value of $\eta$ and $\varphi _\mathrm {th}$, which would indicate that avalanches in this model are compact^29^.

## Discussion

Behavioral cascades, taking the form of intermittent rearrangements (avalanches) in the patterns of movement are an important, albeit sometimes neglected, feature of collective motion in animals. Here we have shown that behavioral cascades can be observed in the rearrangement dynamics of swimming fish schools. Such avalanches, defined in terms of a turning threshold for the heading of the fish, have distributions of sizes and times exhibiting a scaling behavior compatible with a power-law tail truncated by a cut-off that is an increasing function of the turning threshold. A data collapse analysis allows to determine the exponents characterizing the scaling form. We conjecture that such avalanche behavior can be due to the presence of effective leadership in the schools. In order to support this conjecture, we introduce a measure of leadership, based in the concept of avalanche initiators, and observe that, indeed, some fish have consistently an unusually large probability to initiate any avalanche in which they participate. These predominant initiators can be interpreted as effective leaders, determining the start of sudden rearrangements of the school headings. Leadership in the context of avalanche initiation could account for individuals having sudden behavioral changes or specific information about the environment, such as the proximity of a wall.

To check whether the presence of leaders is enough to induce avalanche behavior in collective motion, we have considered a very simple model, consisting in a variation of the classical Vicsek model with the addition of a global leader, which influences the movement of all other particles, subject to random changes in its heading. Interestingly, this simple model displays an intermittent behavior qualitatively similar to that observed in real schools, with avalanche size and duration distributions displaying a self-similar scaling form.

Our results provide a new perspective on the avalanche behavior observed in real collective motion situations^10^, which can be associated to a simple mechanism of leadership that exerts a long range influence, observed in many natural situations, indicating the possibility of a direct relation between these phenomena. Leadership in the present context of a moving school corresponds to those individuals that first react to any external input, or that first exhibit a random behavioral change, and preferentially start sudden rearrangements of the trajectories of other fish in the school. Such interpretation is validated by the numerical results from our model. It is also worth emphasizing that, while it does not offer a perfect quantitative prediction of the characteristic exponents, it nevertheless allows to reproduce the scaling form of the avalanche distributions within a minimalist modeling framework.

Different venues of future research stem from the results presented here. From an empirical perspective, it would be interesting to further study the nature of the avalanches observed in real schools, and to correlate them with other physical properties measured in similar systems^43,44^, as well as with other measures of leadership devised in other contexts of collective motion^38^. From a numerical point of view, our results present new challenges in the understanding of the properties of the proposed model. Indeed, a clearly open question remains to ascertain the ultimate origin of the scaling behavior observed in avalanches in a system in which no apparent critical transition exists. Another interesting question regards the effects of leader switching strategies. We expect the scale-free nature of the observed avalanches to be preserved, provided that the influence of the leader, sensory wise, remains rather long-ranged. In this sense, as we have numerically checked (data not shown), a short-ranged leader, only with local influence over its nearest neighbors, is not able to induce system-wide orientation rearrangements. On the other hand, the value of the exponents associated to the size and duration cutoffs are apparently independent of the noise intensity imposed on the system. These observations hint towards a possible partial universality, which is not shared, however, by the power-law decay exponent. Further work in this direction is clearly needed in order to clarify these issues.

## Methods

### A null model of fish avalanches

In the absence of any sort of dynamical correlations between the turning angles of fish, the evolution of avalanches is purely determined by the independent turning probability $P(\varphi )$ of each fish. As a null model of avalanche behavior, we consider the case in which each fish independently turns an angle $\varphi$ at each time step. Consider an avalanche of duration *t* and size *s*, starting at time $t'=1$. If the avalanche lasts *t* time steps, it means that at least one fish turned an angle larger than $\varphi _\mathrm {th}$ every frame from $t'=1$ to $t'=t$, and that no fish turned an angle larger than $\varphi _\mathrm {th}$ at frame $t' = t+1$. Under these conditions, the probability that a fish turns an angle larger than $\varphi _\mathrm {th}$ in any frame is

9 $$\begin{aligned} q = \int _{\varphi _\mathrm {th}}^{\pi } P(\varphi ) \; d\varphi , \end{aligned}$$

and the probability that at least one fish turns an angle larger than $\varphi _\mathrm {th}$ in a given frame is

10 $$\begin{aligned} Q = 1 - (1-q)^N, \end{aligned}$$

where *N* is the number of fish. Therefore, the normalized probability that an avalanche lasts for *t* frames in this null model is

11 $$\begin{aligned} P_0(t) = \frac{( 1- Q ) Q^t}{\sum _{t' =1}^\infty ( 1- Q ) Q^{t' }} = (1-Q) Q^{t-1}, \qquad t = 1, 2. \ldots , \infty , \end{aligned}$$

where we consider that avalanches have a minimal duration of one frame. That is, in the uncorrelated null model, the avalanche duration distribution has an exponential form, with average avalanche duration $\langle {t}\rangle _0 = \sum _{t=1}^\infty t P_0(t) = 1 / (1-Q)$

Consider now a frame in an avalanche of finite duration. In this frame, at least one fish turned an angle larger than $\varphi _\mathrm {th}$, therefore the probability of observing $s_1$ large turns in this frame is

12 $$\begin{aligned} p_1(s_1) = \frac{1}{Q}\left( {\begin{array}{c}N\\ s_1\end{array}}\right) q^{s_1} (1-q)^{N -s_1}, \qquad s_1 = 1, 2, \ldots , N, \end{aligned}$$

If the avalanche has duration *t*, at each frame a number $s_1$ of fish, distributed with the probability Eq. (12), will turn a large angle. Therefore, the distribution of sizes in avalanches of duration *t*, $P_0(s | t)$ will be given the convolution of the probability Eq. (12) *t* times with itself. The form of this expression is hard to compute. However, we can approximate the avalanche size distribution as follows: Since Eq. (12) is similar to a binomial distribution, it is bell-shaped and centered at the average value

13 $$\begin{aligned} \bar{s}_1 = \sum _{s_1=1}^N s_1 p_1(s_1) = \frac{Nq}{Q}. \end{aligned}$$

Therefore, the average size of an avalanche of duration *t* is

14 $$\begin{aligned} \bar{s}_t = \frac{Nq}{Q} t, \end{aligned}$$

linear with *t*. Assuming that the relation between size *s* and duration *t* is tight, given the bounded distribution $p_1(s_1)$, we can use relation $s \simeq \frac{Nq}{Q} t$ and the distribution $P_0(t)$ from Eq. (11) to obtain the probability transformation $P_0(t) dt = P_0(s)ds$, leading to

15 $$\begin{aligned} P_0(s) \simeq \frac{1-Q}{Nq} e^{- s Q \ln (1/Q)/(N q)}, \end{aligned}$$

that is, an exponential decay with a characteristic size

16 $$\begin{aligned} s_c = \frac{Nq}{Q \ln (1/Q)}. \end{aligned}$$

### Numerical data collapse analysis

We start from a set of avalanche size (or duration) distributions, that we assume to fulfill the scaling relation, at the level of the cumulated distributions,

17 $$\begin{aligned} P_c(s) = s^{-\tau _s + 1} F_s\left( s \varphi _\mathrm {th}^{\sigma _s}\right) . \end{aligned}$$

In order to find the exponents $\tau _s$ and $\sigma _s$, we proceed as follows: We consider general exponents $x_s$ and $y_s$, from which we can write the new rescaled expressions

18 $$\begin{aligned} \varphi _\mathrm {th}^{y_s ( 1 - x_s )} P_c(s) = F'_s\left( s \varphi _\mathrm {th}^{y_s}\right) , \end{aligned}$$

where $F'_s(z) = z^{-\tau _s +1} F_s(z)$. Plotting $\varphi _\mathrm {th}^{y_s ( 1 - x_s )} P_c(s)$ as a function of $s \varphi _\mathrm {th}^{y_s}$, the curves for different values of $\varphi _\mathrm {th}$ will collapse onto the universal function $F'_s(z)$ when $x_s = \tau _s$ and $y_s = \sigma _s$. We can estimate these exponents by considering the difference of the curves for the different values of $\varphi _\mathrm {th}$ and choosing the exponents $\tau _s$ and $\sigma _s$ as the values of the exponents $x_s$ and $y_s$ that minimize this difference. To compute the difference, we locate the interval of values of $s \varphi _\mathrm {th}^{y_s}$ common for all $\varphi _\mathrm {th}$. In this interval, we compute a spline of order *k* for each quantity $\varphi _\mathrm {th}^{y_s ( 1 - x_s)} P_c(s)$ and interpolate a fixed number *n* of equispaced points. The difference is defined as the sum of the variances of the values of $\varphi _\mathrm {th}^{y_s ( 1 - x_s )} P_c(s)$ in each point of the interpolation, for the different values of $\varphi _\mathrm {th}$. In the results presented here, we consider splines of order $k=2$ and interpolate $n=10$ points for each $P_c(s)$ curve.

### Leadership probability in the null model of avalanche behavior

In the avalanche null model defined above, consider a fish that participates in a given avalanche. To estimate its leadership probability we have to compute the probability that it leads the avalanche (i.e. it is active in its first time step), provided that it participates in it. To compute it, we use Bayes’ theorem to write

19 $$\begin{aligned} P(p) P(l | p) = P(l) P(p | l), \end{aligned}$$

where *P*(*p*) is the probability that the fish participates in a given avalanche, *P*(*l*|*p*) the probability of leading an avalanche in which it participates (the probability we are seeking), *P*(*l*) the probability of leading an avalanche, and *P*(*p*|*l*) the probability that a fish participates in an avalanche provided that it leads it. Obviously, $P(p | l) = 1$. To estimate the rest of probabilities, we need information about the duration *t* of the avalanche. Thus, we have $P(p) = 1 - (1 - q)^{t}$, the probability that the fish turns at least once in the development of the avalanche, and $P(l) = q$, the probability that the fish is active (performs a large turn) in the first time step of the avalanche. Therefore, from Eq. (19) we obtain

20 $$\begin{aligned} P(l | p) = \frac{P(l) P(p | l)}{P(p)} = \frac{q}{1 - (1-q)^t}. \end{aligned}$$

Within this null model, consider a fish that participates in $N_a$ avalanches, each of duration $t_\alpha$, $\alpha = 1, \ldots , N_a$. The probability of leading any of these avalanches is $p_\alpha = q / [1 - (1-q)^{t_\alpha }]$. Therefore, the probability $P(\ell )$ of leading $\ell$ of the $N_a$ avalanches is given by a Poisson binomial distribution, representing the probability distribution of a sum of independent Bernoulli trials that have different success probabilities $p_\alpha$^45^. The Poisson binomial distribution has a rather convoluted form, but its mean and variance can be easily expressed as

21 $$\begin{aligned} \mu = \sum _\ell \ell P(\ell ) = \sum _\alpha p_\alpha , \qquad \sigma ^2 = \sum _\ell \ell ^2 P(\ell ) - \left[ \sum _\ell \ell P(\ell ) \right] ^2 = \sum _\alpha p_\alpha (1-p_\alpha ). \end{aligned}$$

The average leadership probability of a fish in this null model is thus given by $\chi _0 = \langle {\ell }\rangle / N_a$, where $\langle {\ell }\rangle = \sum _\alpha p_\alpha$ is the average number of avalanches led by the fish. In Fig. 3 we show the actual values of $\chi _i$ computed for each fish. The full line and shaded region represents the null-model average value $\chi _i^0$ computed for each fish, taking into account the number of avalanches in which it participates, and its $99\%$ confidence interval, respectively.

### Vicsek model with leadership

In the classic Vicsek model^28,39^, *N* self-propelled particles (SPPs) move in a two dimensional space. The dynamics is overdamped and defined in discrete time, with the instantaneous position $\mathbf {r}_i(t)$, $i=1, \ldots , N$, of each particle being related with its velocity $\mathbf {v}_i(t)$ by

22 $$\begin{aligned} \mathbf {r}_i(t+ \Delta t) = \mathbf {r}_i(t) + \mathbf {v}_i(t) \Delta t, \end{aligned}$$

where $\Delta t$ is an integration time step, arbitrarily fixed to $\Delta t = 1$. Velocities have a constant modulus, $|\mathbf {v}_i(t)| = v_0$, and thus are fully determined by their direction, given by the heading angle $\theta _i(t)$ that the velocity forms with, say the *x* axis, such that $\mathbf {v}_i(t) = (v_0 \cos \theta _i(t), v_0 \sin \theta _i(t))$. Heading is assumed to belong to the interval $[-\pi , \pi ]$. In this model each particle *i* tends to orient its direction of motion along the average direction $\mathbf {V}_i(t)$ of the particles located inside a circular area $\mathcal {V}_i$ of radius *R* centered at its own position and including itself, i.e.

23 $$\begin{aligned} \mathbf {V}_i(t) = \frac{1}{n_i(t)}\sum _{j \in \mathcal {V}_i} \mathbf {v}_j (t), \end{aligned}$$

where $n_i(t)$ is the number of particles in the neighborhood $\mathcal {V}_i$ at time *t*. This dynamics is implemented in the update rule for the heading angle

24 $$\begin{aligned} \theta _i(t +\Delta t) = \Theta \left[ \mathbf {V}_i(t) \right] + \eta \; \xi _i(t), \end{aligned}$$

where the function $\Theta [\mathbf {V}]$ represents the angle of vector $\mathbf {V}$ and $\xi _i(t)$ a random noise, uniformly distributed in the interval $[-\pi , \pi ]$, and $\eta \in [0,1]$ a parameter gauging the strength of the noise term.

This model exhibits an order-disorder phase transition defined in terms of an order parameter (polarization) given by

25 $$\begin{aligned} \phi (\eta ) = \frac{1}{v_0 N}\left\langle \left| \sum _{i=1}^N \mathbf {v}_i(t) \right| \right\rangle _t, \end{aligned}$$

the brackets representing a temporal average. The transition separates an ordered phase for noise strength smaller than a critical value $\eta _c$, corresponding to a flocking (schooling) phase, from a disordered phase for $\eta > \eta _c$, corresponding to a swarming, disordered phase.

In the variation of the Vicsek model we consider, one of the SPPs, say particle 1 plays the role of a leader which influences the orientation of the rest of SPPs in the system, independently of their relative distance. Therefore, in the heading update rule Eq. (24), the average velocity of the neighbors is replaced by the average $\mathbf {V}_i^L(t)$ computed in the set $\mathcal {V}_i^{L} = \mathcal {V}_i \; \cup \; \{ 1 \}$, including the global leader and all the particles in the local neighborhood of *i*. The heading and velocity of this leader is constant in time, $\theta _1(t) = \theta _L$, and it represents a privileged direction it wants to follow.

Simulations of the model are performed in square boxes of different size *L* with periodic boundary conditions. We fix the density of particles $\rho = N / L^2 = 1$, the radius of interaction $R=1$, and the constant speed of the SPPs $v_0 = 0.03$.

### Moments analysis technique

The finite-size scaling (FSS) method^41^ assumes that the dependence on system size *L* of the avalanche size and time distributions is of the form

26 $$\begin{aligned} P(s, L)= & {} s^{-\tau _s} \mathcal {F}_s\left( \frac{s}{s_c(L)}\right) , \end{aligned}$$

27 $$\begin{aligned} P(t, L)= & {} t^{-\tau _t} \mathcal {F}_t\left( \frac{t}{t_c(L)}\right) , \end{aligned}$$

where $\mathcal {F}_x(z)$ are scaling functions that are approximately constant for $z < 1$, and decay very fast to zero for $z>1$. The quantities $s_c(L)$ and $t_c(L)$ are the cut-off characteristics size and time, which are assume to depend on system size as $s_c(L) \sim L^D$ and $t_c(L) \sim L^z$, thus defining the standard critical exponents $\tau _s$, $\tau _t$, *D* (the fractal dimension) and *z* (the dynamic critical exponent)^29^.

Assuming the scaling form given by Eqs. (26) and (27), we can compute numerically the associated critical exponents applying the moment analysis technique^42^. One starts by defining the *q*-th moment of the avalanche size distribution on a box of size *L* as

28 $$\begin{aligned} \langle {s^q}\rangle _L= & {} \sum _s s^q\; P(s, L) \simeq \int ds\; s^{-\tau _s + q} \mathcal {F}_s\left( \frac{s}{L^D}\right) \nonumber \\= & {} L^{D(q+1-\tau _s)} \int dx\; y^{-\tau _s + q} \mathcal {F}_s(x) \sim L^{\sigma _s(q)}, \end{aligned}$$

where we have introduce the FSS form in Eq. (26), and taken the continuous approximation for the *s* and *t* variables. The exponents $\sigma _s(q) \equiv D(q+1-\tau _s)$ can be estimated as the slope of the numerical evaluation of $\langle {s^q}\rangle _L$ as a function of *L* in a double logarithmic plot. Then, for sufficiently large values of *q*, we can perform a linear fit of the exponent $\sigma _s(q)$ to the form

29 $$\begin{aligned} \sigma _s(q) = A q + B, \end{aligned}$$

with $A = D$ and $B = D(1-\tau _s)$, from where *D* and $\tau _s$ can be directly estimated. Along the same lines, the exponents associated to the avalanche time distribution can be evaluated considering the *q*-th moment of the time distribution, $\langle {t^q}\rangle _L \sim L^{\sigma _t(q)}$, with $\sigma _t(q) \equiv z(q+1-\tau _t)$.

## Supplementary Information

Supplementary Video 1.

Supplementary Information.

## Data Availability

The datasets used in this study are available from the corresponding authors upon reasonable request.
