# Supplementary material for: Scale-free behavioral cascades and effective leadership in schooling fish
Source: Sci Rep. 2022 Jun 24;12:10783. doi: 10.1038/s41598-022-14337-0 (PMC9232620; doi:10.1038/s41598-022-14337-0)
Supplement: Supplementary file 2 — Supplementary Information. [file 41598_2022_14337_MOESM2_ESM.pdf]

SUPPLEMENTARY INFORMATION FOR
Scale-free behavioral cascades and effective leadership in schooling fish

Julia Múgica,[1] Jordi Torrents,[1,2] Javier Cristín,[3,4] Andreu Puy,[1] M. Carmen Miguel,[2,5] and Romualdo Pastor-Satorras[1]

[1] *Departament de Física, Universitat Politècnica de Catalunya, Campus Nord B4, 08034 Barcelona, Spain*

[2] *Departament de Física de la Matèria Condensada, Universitat de Barcelona, Martí i Franquès 1, 08028 Barcelona, Spain*

[3] *Istituto Sistemi Complessi, Consiglio Nazionale delle Ricerche, UOS Sapienza, 00185 Rome, Italy*

[4] *Dipartimento di Fisica, Universita' Sapienza, 00185 Rome, Italy*

[5] *Universitat de Barcelona Institute of Complex Systems (UBICS), Universitat de Barcelona, Barcelona, Spain*

SUPPLEMENTARY VIDEOS

Supplementary Video SV 1: Video showing the school evolution in time in a segment of series A, between frames 7883 and 7950, in which an avalanche of size $s = 62$ and duration $t = 23$ takes place, for a turning threshold $\varphi_{\rm th} = 0.5$. Red arrows indicate the heading of an active fish in a given frame. Orange arrows indicate the heading of fish that have participated in the avalanche. Blue arrows indicate the heading of non active fish. The animated plot in the right bottom corner represents the average speed of the flock (red line) and its polarization (blue line) as a function of time. In this plot, the region shaded in orange indicates the time span of the avalanche.

SUPPLEMENTARY FIGURES

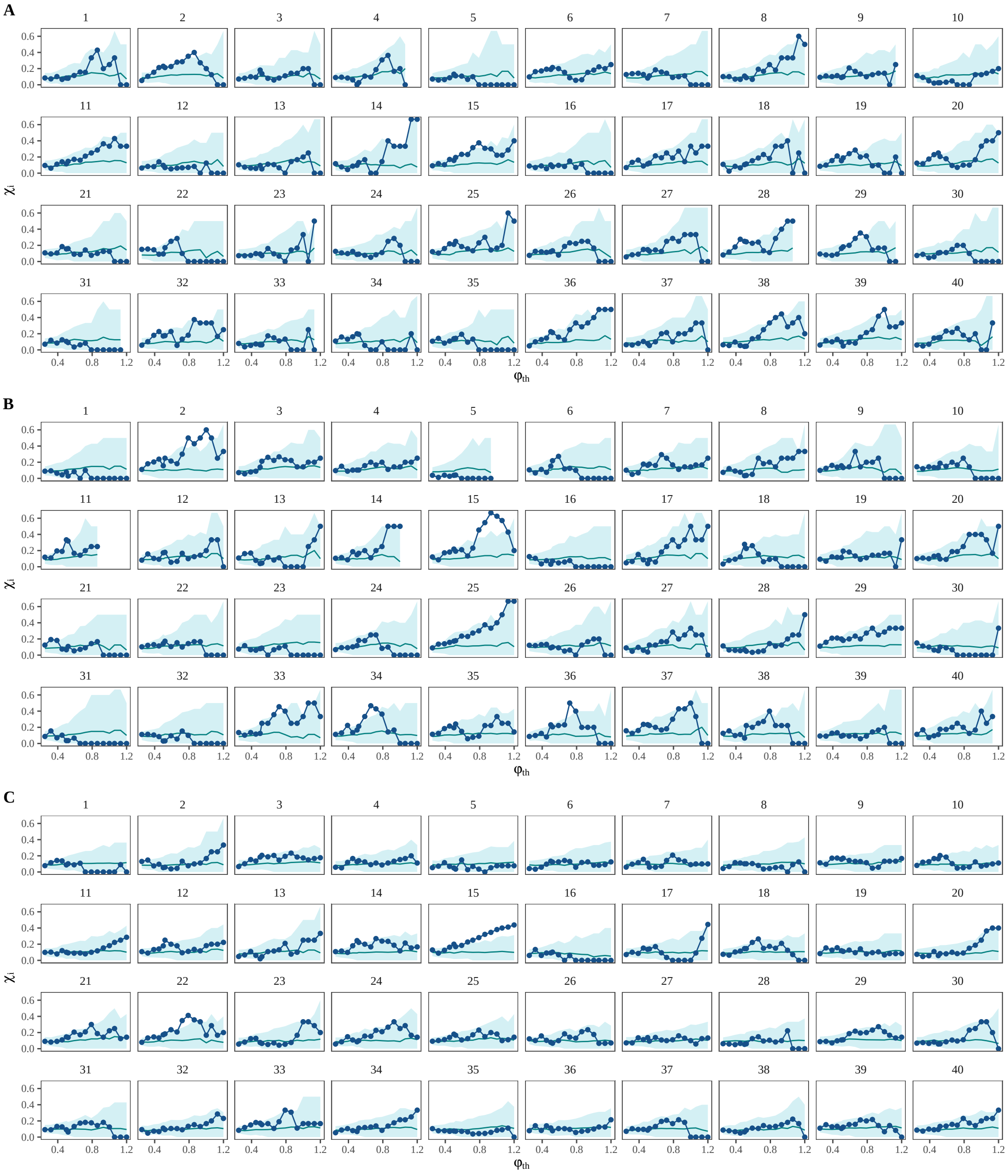

Supplementary Figure SF1. Plot of the leadership probability χ_i as a function of the turning threshold $\varphi_{\rm th}$, for the different fish in each series A, B, C (top to bottom). Full lines represent the average leadership probability in a null model of uncorrelated avalanches. The shadowed regions represents the 99% confidence interval of this value. Notice that in some plots certain values of $\varphi_{\rm th}$ are missing. This is due to the fact that the corresponding fish do not participate in any avalanche, and therefore its leadership probability is not defined.

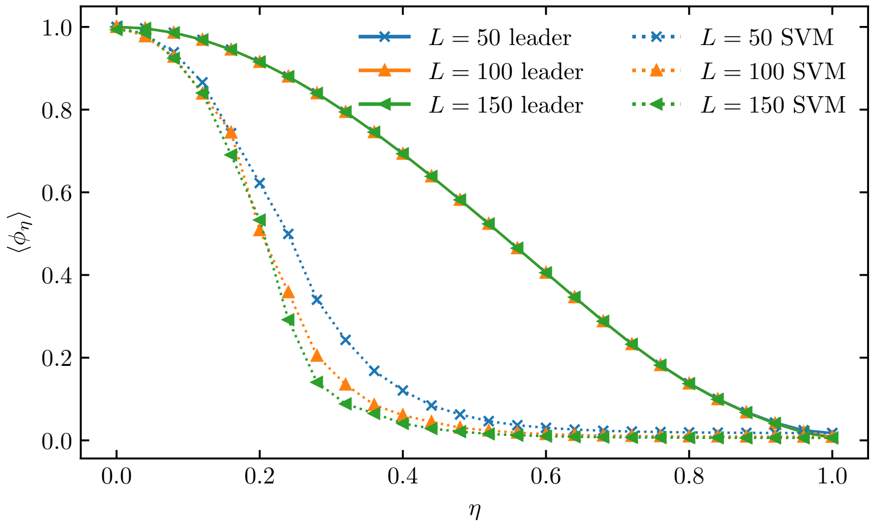

Supplementary Figure SF2. Average order parameter $\langle \phi(\eta) \rangle$ as a function of noise intensity in the classic standard Vicsek model (SVM) and the Vicsek model with a non-rotating global leader for different system sizes.

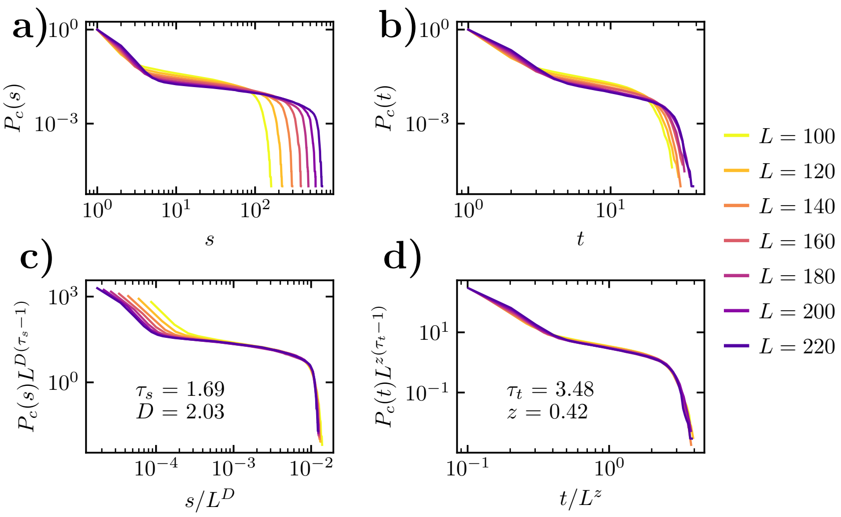

Supplementary Figure SF3. (a) Cumulative probability distribution of size $P_c(s)$ of avalanches induced by a rotating leader in a system with $\eta = 0.3$, turning threshold $\varphi_{\rm th}(\eta)$ and different values of L. (b) Cumulative probability distribution of durations $P_c(t)$ of avalanches induced by a rotating leader in a system with $\eta = 0.2$, turning threshold $\varphi_{\rm th}(\eta)$ and different values of L. In both cases, statistics is performed over at least 10^5 different avalanches. (c) Check of the scaling of the cumulated size distribution with turning threshold $\varphi_{\rm th}(\eta)$, as given by Eq. (8) in the main paper. (d) Check of the scaling of the cumulated time distribution with turning threshold $\varphi_{\rm th}(\eta)$, as given by Eq. (8) in the main paper.

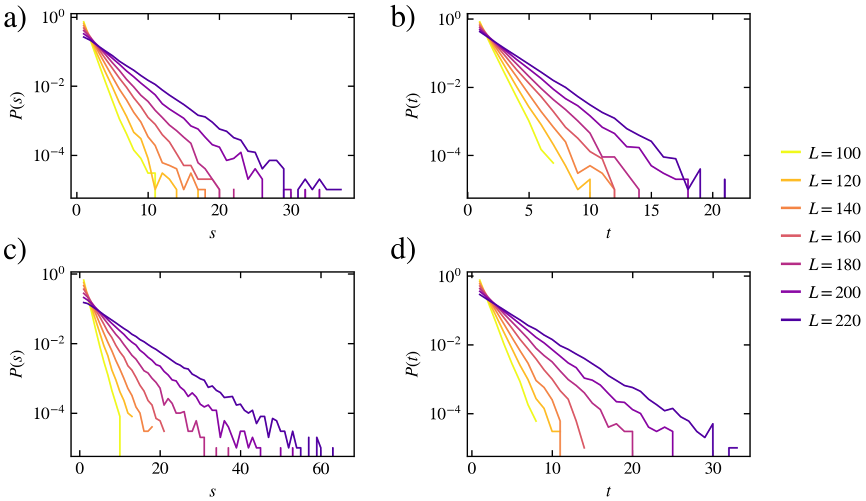

Supplementary Figure SF4. (a) Cumulative probability distribution of size $P_c(s)$ of avalanches in the standard Vicsek model with $\eta = 0.2$, turning threshold $\varphi_{\mathrm{th}}(\eta)$ and different values of L. (b) Cumulative probability distribution of duration $P_c(t)$ in the standard Vicsek model with $\eta = 0.2$, turning threshold $\varphi_{\mathrm{th}}(\eta)$ and different values of L. (c) Cumulative probability distribution of size $P_c(s)$ of avalanches in the standard Vicsek model with $\eta = 0.3$, turning threshold $\varphi_{\mathrm{th}}(\eta)$ and different values of L. (d) Cumulative probability distribution of duration $P_c(t)$ in the standard Vicsek model with $\eta = 0.3$, turning threshold $\varphi_{\mathrm{th}}(\eta)$ and different values of L. Statistics are performed over at least 10^5 different avalanches.

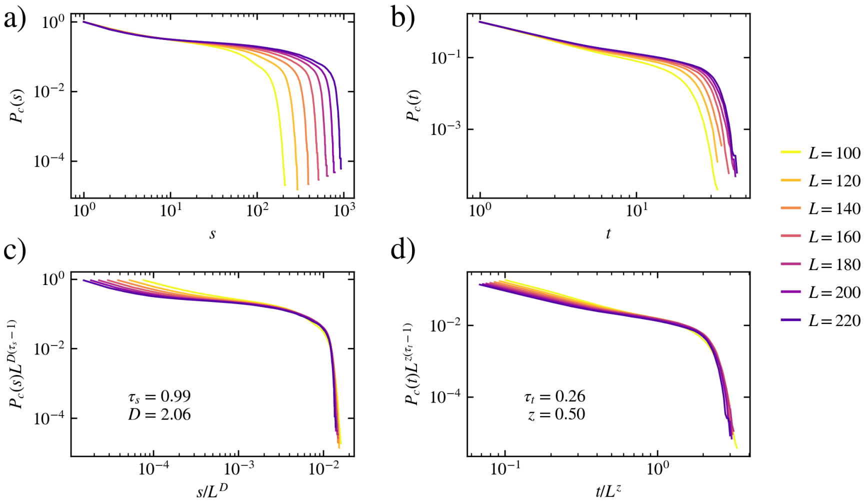

Supplementary Figure SF5. (a) Cumulative probability distribution of size $P_c(s)$ of avalanches induced by a rotating leader in a system with $\eta = 0.2$, turning threshold $\varphi_{\rm th} = 2.8\pi\eta$ and different values of L. (b) Cumulative probability distribution of durations $P_c(t)$ of avalanches induced by a rotating leader in a system with $\eta = 0.2$, turning threshold $\varphi_{\rm th}(\eta)$ and different values of L. In both cases, statistics is performed over at least 10^5 different avalanches. (c) Check of the scaling of the cumulated size distribution with turning threshold $\varphi_{\rm th}(\eta)$, as given by Eq. (8) in the main paper. (d) Check of the scaling of the cumulated time distribution with turning threshold $\varphi_{\rm th}(\eta)$, as given by Eq. (8) in the main paper.

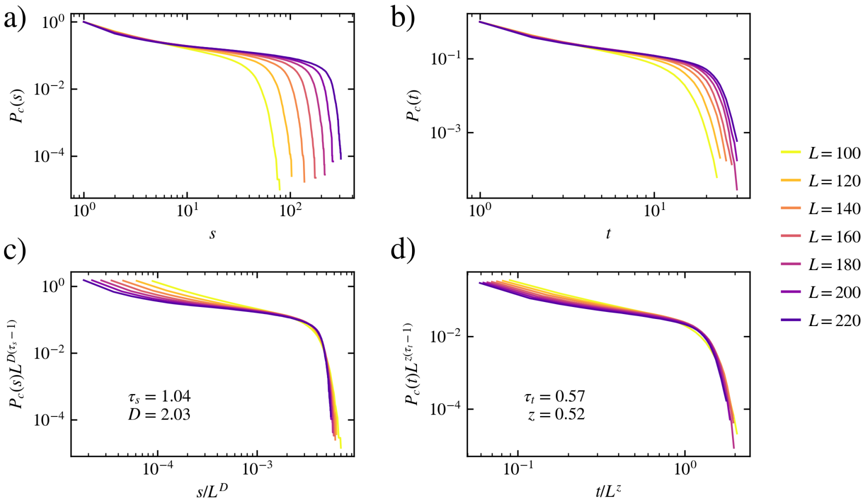

Supplementary Figure SF6. (a) Cumulative probability distribution of size $P_c(s)$ of avalanches induced by a rotating leader in a system with $\eta = 0.3$, turning threshold $\varphi_{\rm th} = 2.8\pi\eta$ and different values of L. (b) Cumulative probability distribution of durations $P_c(t)$ of avalanches induced by a rotating leader in a system with $\eta = 0.3$, turning threshold $\varphi_{\rm th}(\eta)$ and different values of L. In both cases, statistics is performed over at least 10^5 different avalanches. (c) Check of the scaling of the cumulated size distribution with turning threshold $\varphi_{\rm th}(\eta)$, as given by Eq. (8) in the main paper. (d) Check of the scaling of the cumulated time distribution with turning threshold $\varphi_{\rm th}(\eta)$, as given by Eq. (8) in the main paper.
